# Hospital and Institutional News

**Published:** 1913-01-04

**Authors:** 


### HOSPITAL AND INSTITUTIONAL NEWS.

### OUR CHRISTMAS FESTIVITIES SECTION.

In order to fully appreciate the accounts of "Christmas in the Hospitals," which form the most prominent section of The Hospital this week, one or two points must be borne in mind. Each account has been written from inside, in the large majority of instances by the member of the staff who bore the chief part in the arrangements; and no importance attaches to the place of any institution on the list. Our achievement is to give a bird's-eye view of Christmas all over the country, and for this reason institutions have been juxtaposed of diverse types and often from the opposite ends of the country. No preference has been given to size or importance, for it is the character of each institution which Christmas seems to bring out, and the hospital spirit is quite as important in the least-known institution as in the greatest. Another point is worth notice. Some of the accounts naturally form better reading than others, and here, again, every institution, small as well as great, has a perfectly equal chance of making the best impression. The amount of variegated ideas for decorations and entertainments is astonishing, and the wealth of suggestion which these accounts reveal would be enough alone to justify their numbers. The record which we present this week is probably unique, and it is an example of what The Hospital's readers can do when they really take pains to exchange their experience in our columns.

### THE CRUX OF THE CRISIS.

As we go to press the state of confusion over the administration of medical benefit seems to be worse than ever. The Radical Press jubilantly asserts that Mr. George has beaten the British Medical Association all along the line; the Unionist organs are just as cocksure that it is the doctors who are winning. The thing certain is that medical benefit is now to be an affair of party politics, which was the one fate that should have been avoided at all costs in the interest of the insured. For this lamentable condition each side will, of course, blame the other; but the medical profession is not accustomed to mix business and politics, and entertains no doubt at all that the importation of political colour into the negotiations is entirely the work of the author of the Act. His professions of benevolence and appreciative sympathy with the doctors a year and a half ago ought to have put them on their guard; but medical men are slow of suspicion. All the appearances point to a serious split in the Association; and to this division of forces the Chancellor will certainly owe his victory, if victory he eventually achieves. The one point to grasp is that the exigencies of party and not the doctors are causing all existing rancour, opposition, and ill-will. If the Government were not compelled by political considerations to squeeze through certain highly controversial measures, for which purposes the Insurance Act must first be "got out of the way," the Chancellor would soon have found reasons for giving further time to consider contentious points. As it is, time is denied for the sake of a congested programme, and facts are revenging themselves like hornets wantonly disturbed.

### THE NEW YEAR'S OPPORTUNITY.

No reader will fail to appreciate the splendid response which The Hospital's invitation for accounts of Christmas festivities has received. As far as space would permit, and some senders, we fear, will be disappointed, every quarter of the country and every type of institution is represented in the charmingly diversified accounts that we publish elsewhere this week. This is exceedingly gratifying for two reasons. In the first place, it has enabled us to place on record what the voluntary hospital spirit, which is seen in its most typical aspect at Christmas, really achieves, and what a true blessing and living force for good it is; and secondly, it shows that our readers fully appreciate the fact that The Hospital is their paper, whose function is the twofold one of supplying them with expert information in grave difficulties or times of crisis, and that our columns are also the channel by which the inside life and economy of our institutions are made known to the external world. As co-operation is the keynote on which a well-conducted institution depends, so it is natural that co-operation should be well understood in the wider sense by institutional workers. It is to further manifestations of this co-operation between readers and The Hospital, which has produced the fine descriptive array of hospital Christmas festivities this week, that we look forward during the New Year. This is the note that we wish to strike at its commencement. Every institution has some phase of interest, some new development, or scrap of history, and every week brings out in some institution that individual character and message which Christmas makes the opportunity of all. We therefore ask our readers to do what they have done this week during each of its successors—that is, to use The Hospital as the common meeting place and centre of solidarity. In this way it will be the expression of every side of hospital life, for, as a great editor once remarked, a newspaper is just as good as its readers choose to make it.

### THE NEW YEAR HONOURS.

It might almost be said that medical men were conspicuous by their absence in the New Year Honours List were it not that the whole hospital world will congratulate Robert William Philip, M.D., F.R.S. (Edinburgh), on his knighthood. As senior physician to the Royal Victoria Hospital for Consumption, Edinburgh, for the establishment of which, with its farm colony, he was largely responsible so far back as 1887, he well deserves the honour. The Local Government Board for Scotland has since adopted Sir Robert's as a national system for the administration of the anti-phthisis campaign, and his works on the etiology and treatment of consumption have made this recognition, tardy as it is, inevitable, and we hope that a knighthood will not be the full measure of public regard for the debt which is certainly owed to Sir Robert Philip. Mr. James Key Caird, who has endowed a tuberculosis and cancer hospital at Dundee, receives a baronetcy. Under the medical knights we may be forgiven for including Dr. Francis Darwin, for though he is a Doctor of Science, not of Medicine, it was the study of medicine at St. George's which led him to assist Charles Darwin in his work. The Knight Commandership of the Bath is conferred on Inspector-General of Hospitals and Fleets Duncan Hilston, C.B., M.D., retired, whose last appointment was the charge of Haslar Hospital.

### THE INDIAN LIST.

Passing then to the Indian List, we note that the Companionship of the Order of the Indian Empire, C.I.E., is bestowed on Major William Glen Liston, M.D., D.P.H., Indian Medical Service, who is the Director of the Bacteriological Laboratory, Parel, and also the Senior Member of the Plague Research Commission; and on Lieutenant-Colonel Walter James Buchanan, M.D., Indian Medical Service, Inspector-General of Prisons, Bengal. The "Kaisar-i-Hind Medal for Public Service in India" of the First Class is awarded to Major Henry William Grattan, Royal Army Medical Corps, Officer-in-Charge, Enteric Fever Convalescent Depôt, Naini Tal; to Dr. John Andrew Turner, M.D., C.M., D.P.H., Health Officer, Bombay Municipality; to Major Ernest Reinhold Rost, Indian Medical Service, Senior Civil Surgeon, Rangoon, Burma; and to Major Ellacott Leamon Ward, Indian Medical Service, Punjab. Finally, David Hardie, M.D., of Brisbane, and George Turner, M.B., Medical Superintendent of the Pretoria Leper Asylum, have received the honour of Knight Bachelor.

### ARTIFICIAL VENTILATION IN CINEMA THEATRES.

Our description last week of the cinematograph entertainment as an *enfant terrible* in many spheres did not go far enough. The list then given of the problems which it has created must be supplemented by a new one, to which attention is drawn by Dr. Jones, the County Medical Officer for Surrey. He points out in his annual report the risk of spreading infectious disease which exists in all adapted buildings, and especially in cinematograph theatres, which are not only as a rule adaptations, but also as a class, and perhaps partly for this reason, very badly ventilated. Dr. Jones places the genuineness of the risk beyond cavil when he remarks so acutely that the problem of ventilation is doubly difficult in a building from which all light must be excluded. He therefore argues that inadequate natural means of ventilation should be reinforced by mechanical methods. Now the question of mechanical ventilation itself is one which is frequently engaging the attention of institutional authorities. For though the best opinion is almost unanimously in favour of natural methods of securing change of air and relays of air-currents, certain artificial systems, like the "Plenum," have strong supporters, who are entitled to receive attention. Hospitals, then, would have cause to be grateful if Dr. Jones' remarks on the bad natural ventilation of the cinematograph theatre should lead to further experiments with artificial methods, or, if certain of our readers prefer it, to the production of further proofs of the value of already existing mechanical systems. Competition here might prove useful, and, if it did so, the cinematograph theatre, where the problem is far more acute than in any hospital, might be the means of providing useful lessons for hospital architects and boards of management.

### A HOSPITAL EXHIBITION.

In addition to the details of the coming International Medical Congress to be held in London already published in The Hospital, attention should be specially directed to the technical exhibition which is being organised. It will be held in the great hall and the two smaller halls of the University of London, Imperial Institute, and will illustrate hospital design, construction, arrangement, and equipment; surgical and medical instruments and appliances; sanitary appliances for the ward; pharmaceutical preparations, disinfectants, and dietetic articles; microscopes and other apparatus used either in pathology, bacteriology, or in clinical instruction; appliances for the treatment of the sick and wounded in war, and emergency aids for street accidents, etc.; and carriages and other personal requirements in professional practice. Awards for the best exhibits in each section will be given; the judges will be an independent subcommittee of well-known medical men; and any information can be obtained from the Exhibition Offices, 194-200 Bishopsgate, London, E.C. Mr. Rutland S. Ely is the secretary. Those who intend to show models or plans of hospital design, construction, and equipment will doubtless turn with confidence and profit to their bound volumes of The Hospital. We trust that such references will result in many awards of honour; and we could also wish that some way might be found of incorporating Mr. Wellcome's historical project with that of the technical exhibition, and of conferring some award upon its originator.

### A QUEEN ON ENGLISH INSTITUTIONS.

Last Saturday afternoon Queen Amelia visited the Royal Hospital, Richmond. It was not one of those informal visits which Her Majesty has often made in order to see, as she has seen many times, the characteristic aspect of everyday institutional life, but it was made in order that she might be present at the Christmas-tree distribution, which took place in the Princess May children's ward. One of the most successful Santa Claus of the year was surely that of Dr. Rhodes, the senior house surgeon, and it is well to emphasise such a point, for people are apt to forget that the figure of Santa Claus is one of such dramatic breadth that practically every personality may find expression in the traditional ritual which he plays, and under the vestments which so many centuries have hallowed. It is, moreover, not a little illuminating to recall that Santa Claus is always the central figure in hospital Christmas, however much he may be relegated to the background in institutions that care less for the spirit of personal service which he typifies, and have less need of his cheering presence than hospitals. It is because he is the incarnation of personal service, because Christmas Eve and Christmas Day are his most hard-working days of all, that he is so invariably beloved by hospital workers. He represents in splendid vestments and on one occasion the spirit and the work which are the bases of hospital life through every day in the year. It was, perhaps, with some such thought in her mind that Queen Amelia remarked to the acting chairman, Colonel J. Leslie Powell, on leaving: "I admire all your English institutions." Of them all the voluntary hospital has become the most typical, and the spirit which inspires them, and that foreign visitors so often admire, is the spirit of which Santa Claus is the symbol.

### A CRITICISM OF THE RADIUM INSTITUTE.

In both the lay and professional press Dr. Warden, physician to the British Hospital in Paris, draws attention to a defect in the organisation of the Radium Institute and proposes a remedy. None of the comparatively large supply of radium at the Institute is allowed to leave the Institute's walls—a precaution against loss by carelessness or dishonesty, which is, we must confess, a wise one. Owing to the fact that the Institute contains no beds, patients who are not sufficiently able-bodied to travel backwards and forwards thither are excluded from all share in its benefits; and for the same reason no one can have radium-containing tubes buried in his tissues by operative means. Thus the Institute is cut off from two large sections of radium therapy, which are being exploited elsewhere with some considerable success. One illustration of this contention will be found by anyone who cares to test it in the *Edinburgh Medical Journal* for December, wherein is given an account of a most successful case of radium therapy in inoperable malignant disease. This case would have had to be refused at the Radium Institute had the patient been a Londoner instead of an Edinburgher. We feel that Dr. Warden's criticism is just, though we understand also the difficulty in which the authorities at Riding House Street are placed. A better solution than that which the Paris physician advocates would be the provision of wards and an operating theatre at the Institute. Then all the uses of radium could be practised without any risk of loss of the precious element.

### A NEW TROPICAL DISCOVERY.

Only last week we commented on the marked success of Mr. Austen Chamberlain's efforts on behalf of the London School of Tropical Medicine. This week the School is able to show a return for the money which has been and is being subscribed to it, for the Wandsworth Scholar, Dr. R. T. Leiper, has telegraphed from Calabar that he has discovered the "intermediate host" of the parasite *Filaria loa*, the cause of "Calabar swelling" and other tropical ailments. It will be remembered that the Scholarship in question was founded about six months ago by the bequest of £10,000 under Lord Wandsworth's will, which sum was to be applied by Sir William Bennett at his absolute discretion for medical research. Sir William handed over the whole sum to the London School, and it has not been long in yielding a notable result. There are two kinds of this parasite *filaria*: one is found in the blood of the patient during the night time only, and is the cause of elephantiasis; the other is found in the blood only during the day time, and does not cause elephantiasis. It was years ago surmised by Sir Patrick Manson that one parasite must be transferred by some nocturnal biting insect and the other by a diurnal feeder. The former view was proved by its originator to be correct and the carriers to be certain species of mosquito. The other half of the hypothesis has so far remained unproved, but Dr. Leiper now announces that a fly of the *Chrysops* genus has been found guilty. This important discovery has come in the nick of time to stimulate public interest and generosity towards Mr. Chamberlain's appeal.

### NEW PRINCIPAL OF HARTLEY COLLEGE.

Dr. Alexander Hill, M.D., F.R.C.S., has been elected Principal of Hartley University College, Southampton, at a salary of £1,000 a year. It is not often that any man combines such a distinguished medical and educational record as Dr. Hill. Downing College, Cambridge, and St. Bartholomew's Hospital were the chief centres of his education, and he repaid them both by becoming Hunterian Professor at the Royal College of Surgeons in 1884-5, only to be appointed Master of Downing in 1888. As Master of Downing he presided till 1907, and it is, no doubt, a tribute to his work there that he has been chosen Principal of Hartley College, which has had to fight to preserve its University status through a period of difficulty. Nearly £32,000 has been raised or promised during the past year, and the first block of a new college will be undertaken, so that in two senses a new era is likely to begin under Dr. Hill. Indeed, Mr. C. G. Montefiore has just forwarded £1,900 to make up the £32,000 required by the Board of Education for the retention of the Exchequer grant. Dr. Hill's many other activities have included membership of the Treasury Commission to report on University Colleges, and he has also held the offices of President of the Neurological Society and of the Teachers' Guild. "Notes on Browning's Poems," "The Plan of the Central Nervous System," "A Run Round the Empire," and a "Primer of Physiology" represent the many sides of his authorship. He has always brought culture to medicine and infused education with science and the spirit of public affairs.

### INCREASED PAY FOR MENTAL HOSPITAL WORKERS.

The London County Council has recently amended the scale of remuneration of various grades of workers at its various mental hospitals. The scale of clerks of mental hospitals, who now begin at £210 a year, rising by annual increases of £10 to £360 a year, with dinner daily when at the hospital, has been altered so as to provide for an annual increment of £15. The scale of remuneration for house stewards, which formerly began at £260, rising by £10 a year to £310, with breakfast, dinner, and tea when at the hospital, has also been amended to provide for a maximum salary of £360 a year, which will be reached by annual increments of £15, except for £10 in the last year. The maximum of the scale of rate of pay for head attendants has been increased from £75 to £80 a year, with board, lodging, washing, and uniform. We are glad to notice the increasing recognition that slowly no doubt, but still surely, is being given to the claims of those engaged in mental work. It has taken a long time for the strain upon every officer in a mental hospital to be officially admitted, on the pay-sheet at least. We welcome, therefore, the action of the London County Council, not only for the alterations which it entails but for the promise that it holds out of more general recognition in the future.

### THE RESIDENTS' PLAY AT GUY'S.

During Christmas week the resident medical staff at Guy's Hospital have been very active, and the excellence of their play points to the arduousness of their rehearsals. Entitled "Progress: or the Light that Failed," a title sufficiently witty to attract any competent critic, the play cleverly portrayed Science from the medical man's point of view, with many good-natured hits at the staff and their pet theories. The very realistic Boro' mother was much applauded, and the get-up was equal to the acting. The piece was so popular that on each of the three evenings the physiological theatre—not the operating theatre, as one of the "dailies" had it—was crowded to its utmost limit, and many old friends came from afar to hear the latest that a topical play can suggest. The residents are to be congratulated on their production and on the very excellent music supplied by various members of the hospital and their friends. We hope that the text has been preserved, if only as a record to show that Mr. Bernard Shaw is not the only playwright who can see the comedy latent in medical science and in its protagonists to-day.

### AN OUT-PATIENT DEPARTMENT FOR MENTAL CASES.

An official announcement has appeared to the effect that an out-patient department for mental diseases is being constituted at University College Hospital, and applications are invited for the post of physician in charge of it. Without the least desire to be hypercritical, we should have thought that a few months' delay in this project might have been advisable in order that the effect of the Insurance Act upon out-patient practice might be realised and considered. Most hospital managers seem to be looking forward to a reduction in out-patient attendances, whatever the result may be in the case of in-patients. Apart from this small point, the experiment should have a good chance of success, which we most cordially wish it. University College Hospital is rather given to innovations of a progressive kind in connection with the medical staff. Some little while ago a special physician to deal with diseases of the heart was appointed. In that case the authorities had at hand the one man for the post in Dr. T. Lewis, who was last year further placed upon the regular staff of assistant physicians. This new departure will be watched eagerly by those who take an active interest in the unsolved problems of the out-patient department.

### RECHRISTENING THE ROYAL SOCIETY!

In the correspondence columns of the *Lancet* Mr. Percy Dunn puts forward the suggestion that the Royal Society of Medicine should seek to change its name to that of the Royal Academy of Medicine. He argues that an academy is a "higher tribunal" than a mere society, and that the phenomenal success of the R.S.M. justifies it in seeking the prestige of this superior title. We do not think Mr. Dunn has made out his case in the least, for we do not admit his premiss that an academy has necessarily a more impressive name than a society. If anyone wants an illustration of this contention we would point to the Royal Society itself, whose two and a half centuries are surely sufficient to refute Mr. Dunn. If he can persuade that august body to change its name it will then be time enough for the Royal Society of Medicine to think of following the example. Young as the latter is, it has already built up such a reputation that any change of name would be unfortunate, unless there were compelling reasons for such a step.

### HOME HEALTH RESORTS.

Not the least important factor in choosing a health resort is the stimulating effect of entirely novel surroundings, and it is often for this reason that English patients are sent abroad. Applied conversely this excuse is perfectly valid in justifying foreign physicians to send their patients to British resorts. Informative literature on the subject of Continental spas abounds and English translations are published broadcast. Now we are glad to be able to direct attention to an authoritative volume, entitled "Health Resorts of the British Islands," which should form a valuable reference book both for British and Continental physicians. The work has been edited by Dr. Neville Wood with the assistance of an advisory committee appointed by the Council of the Section of Balneology and Climatology of the Royal Society of Medicine, and a French edition is now being prepared. Within its covers will be found a fairly exhaustive account of the watering-places, spas, and climatic stations of the British Isles. One of the most interesting chapters is written by the editor and deals with the international aspects of our health resorts. Another useful but somewhat brief chapter is that in which Dr. A. Latham refers to sanatorium treatment, and in which is included a list of sanatoria charging two guineas a week and upwards. Interspersed throughout the book are numerous good photographs and three maps, which latter, however, leave something to be desired on the score of clearness. There is little question that this work, which has just been published by Messrs. Hodder and Stoughton at the hardly popular price of 10s. 6d., will be regarded as an authoritative account of the resources of, and indications for treatment at, our own too much despised health resorts by those medical men and the laity who can afford to buy it.

### THE NEW YEAR'S REFERENCE BOOKS.

The up-to-date hospital secretary has to be such an encyclopædia of knowledge, and to rely on authoritative detail for so many public matters, that a glance at the reference works on his shelf becomes a real test of his keenness and efficiency. It is not only Burdett's *Hospitals and Charities*, which is his Bible, that is needed, but "Who's Who" for its ready biographical information—this year, too, doubly valuable from its increased number of names, and from the thin paper and slightly larger page which has enabled the enlarged volume to retain its comparative thinness. The "Who's Who Year Book" for 1913 is also a useful summary of many more expensive directories and almanacs, while the "Writers' and Artists' Year Book," another of Messrs. Black's publications, is always useful to a publicity department of a hospital. These publishers have also issued a new directory under the title of "Books that Count," price 5s. net. It aims at being a dictionary of standard books, in so far as these are not designed for the specialist only, and it includes a list of good translations from foreign classics and modern works generally that give a good general survey of their subject. The indices refer to authors and titles respectively, and fourteen sections are included under such broad divisions as literature, travel, science, music, and so forth. Its aim is to be the ideal librarian for busy people. Herbert Fry's "London Charities" is a useful eighteenpennyworth, in which Chatto and Windus provide a wonderful amount of statistical information. Equipped with *Burdett* and these allies, every hospital secretary can start the New Year knowing that he has in his office the means of answering without loss of time three-quarters of the questions on public affairs with which he will be confronted so many times during the year.

### THE ADMINISTRATION OF ANÆSTHETICS IN MELBOURNE.

The question of the administration of anæsthetics has recently created a good deal of feeling—and that, too, not of the friendly variety—in the State of Victoria. The trouble arose out of a case at the Melbourne Hospital, where, as a *dernier ressort*, a moribund patient was operated on, and, being an emergency operation, the anæsthetic was administered by one of the resident medical officers. The patient died on the table, and the City Coroner was duly notified. At the inquest this official allowed a Dr. Hornabrook, one of the honorary anæsthetists at the Melbourne Hospital, but who was not present at the operation, to give evidence, or rather to air his opinions, which were to the effect that a senior resident anæsthetist should be on the staff of all large general hospitals. The Coroner, Dr. Cole, returned a verdict that the anæsthetic was necessary, and had been carefully administered; but forwarded Dr. Hornabrook's suggestion to the Government, stamped not only with his approval, but recommending that non-compliance therewith on the part of a hospital should be punished by a reduction or entire withdrawal of the Government grant. The Premier, Mr. Watt, after making inquiries, was satisfied that the statistics neither warranted a scare nor legislative interference, and so the matter drops. But Dr. Hornabrook again came to the fore, and submitted that cases of death after operation should be reported by the anæsthetist, but the board of the Melbourne Hospital has decided to let that responsibility rest, as hitherto, with the medical superintendent.

### THE PROSTITUTION OF A NAME.

Hospitals are sometimes required to undertake very onerous duties as trustees if they benefit by a testator's bequest, and, although that is hardly the case with the Stamford Hospital, its position under Mr. J. G. Dearden's will is worth attention. Mr. James Griffith Dearden, F.S.A., of Walcot House, Northants, who left estate of the gross value of £531,810, has directed that his body should be cremated and the ashes interred at the same place where his dog Pompey is now buried. He further charged his estates in Southorpe with £500 to the Stamford Infirmary if these interments are disturbed or removed within a hundred years. He directed that Walcot Park shall not be sold, exchanged, or otherwise disposed of without and unless the life-tenant shall previously pay or secure the previous payment of £5,000 to the Stamford Infirmary and £5,000 to the Peterborough Infirmary. In effect, as will be seen, the Stamford and Peterborough Infirmaries are employed as a "brake," as if the testator were more anxious to preserve the continuity of his family estates than to subscribe to charity for its own sake. This would be prostituting the name of charity, had not the testator most praiseworthily made his true intentions quite clear.

### £200,000 FOR THE HOSPITALS.

The Honorary Treasurer of the League of Mercy, speaking at St. James's Palace at the annual meeting of Presidents, expressed a wish that every effort should be made to keep up the amount of the contributions this year. He further asked for £1,000 from those members of the public who appreciated at its true value the personal service rendered by members of the League. The response has been most generous; the sum asked for has been sent to the Honorary Treasurer in testimony of the public's appreciation; and the members of the League have since added further contributions to the amounts reported at the annual meeting. The League of

Mercy has thus been enabled to contribute to the voluntary hospitals this year £20,305, making the total amount raised and paid to the hospitals by the League of Mercy, £200,000. This public testimony to the appreciation of the work done by members of the League of Mercy justifies the confidence expressed by the Honorary Treasurer that with such encouragement the results of the members' efforts during 1913 will be more productive than ever.

### WALES AND TUBERCULOSIS.

The interest of King Edward VII. in anti-tuberculosis measures is well known; and it was unanimously conceded that to celebrate his reign, in what was for more than half a century his own Principality, by a far-reaching scheme to minimise the ravages of the "White Plague" there, would be the kind of memorial he himself would most have desired. Accordingly the Welsh Memorial Committee set to work, and accumulated some £200,000 for the purpose, mainly owing to the magnificent liberality of Mr. David Davies, who is said to have given more than half the total sum. Then came the Insurance Act, with its anti-tuberculosis clauses, which obviously had to be taken into account by the Welsh Memorial Committee, in order to avoid overlapping and waste of energy. The Committee decided to become an Approved Society under the Act. This decision has been fiercely criticised by Dr. G. Arbour Stephens, of Swansea, who sees in it an attempt to filch from the very poor the funds subscribed in memory of the late King in order to benefit the well-to-do artisans with whom the Insurance Act is chiefly concerned. According to Dr. Stephens, who lately addressed a meeting of delegates from Welsh Boards of Guardians at Swansea, the result of fusing the Memorial with the Act is to place practically unlimited powers of expenditure and control into the hands of three or four officials who are responsible to nobody for what they do. If the officials in question are of the highest competence and conscientiousness, we think they will probably do better than any popularly elected body. Dr. Stephens seems not to think that they are; but that is a matter into which we cannot follow him. His representations to the assembled delegates were evidently taken to heart, for a committee was at once appointed to confer with the Memorial Association.

### WORKING A FESTIVITIES FUND.

We feel sure that our readers will appreciate the juxtaposition of an article on "The Aim and Working of a Festivities Fund," by the Chairman of the Warrington Infirmary, with the accounts of the festivities themselves, to which it forms the natural preface. No one can fail to be interested in the highly novel and personal appeals which have emanated from the Chairman of this hospital, and have exercised such energies as command respect even from those who best know what voluntary effort can do. It will be seen that the enterprise manifested by Mr. Smethurst is of the genuine quality which makes everything subservient to the end in view, and the result is a batch of new experiences, and a number of new ideas for raising funds for Christmas entertainments, which no up-to-date committee of managers can afford to disregard. Every locality has its own atmosphere, but, in spite of this, each hospital has something to teach as well as to learn from its sister institutions, as is astonishingly well seen from the different hints for decorations or amusements, or for the raising of money, which make half the interest of this article and its immediate successors. The promptness shown by Mr. Smethurst in sending an account of his experiences forms a remarkable example, which we hope every institution will bear in mind. For the limit of perfection is never reached, and the institutions which have shown the way on this occasion are, we hope, only the pioneers of still greater co-operation to come.

### THE REVIVAL OF PRESCRIPTION WRITING.

The New Year is likely to witness a considerable revival in the art of prescription writing. The great extension during the last twenty years of the practice of dispensing among doctors has had the result of making the rapid writing of a pharmaceutically correct prescription a matter of some labour, at any rate among general practitioners. Now that a great deal of the dispensing for insured persons is to be directed into the hands of the chemists, many doctors may find some difficulty in acquiring that impressive fluency in writing their prescriptions so necessary to hold the confidence of the patient. No doubt in many cases the doctors will transport their system of having a certain number of stock mixtures ready into their corresponding pharmacies on the panel, but nevertheless there must be a great increase in the number of prescriptions to be written out in full. Pharmacists may be expected to receive a certain number of weird and incompatible combinations, and on the other hand a certain number of orders for proprietary tablets and preparations, whereby some will seek the easiest path. Doctors have not sufficiently realised the harm to themselves which accrues through the practice of ordering certain, though not all, forms of proprietary medicines. In many cases this method simply means that the patient purchases the drug for himself at a store without further reference to the doctor on every subsequent occasion when he feels in need of treatment. For the Insurance Act's effect on the future of pharmacy our readers are referred to another page.

### THIS WEEK'S DRUG MARKET.

Business in the Drug market has not yet recovered from the Christmas holidays, and until the process of stock-taking is over no improvement can be expected. Few changes in value have occurred since the last report appeared, but on the whole prices are well maintained. The tone of the quinine market is good, but in the case of opium there is a slight tendency towards lower prices. The expected advance in the price of santonin has been announced. Oil of juniper is dearer, and there is a tendency towards higher prices for oil of cloves. Sugar of milk tends lower in value.

